# The Evaluation of Efficacy and Safety of A Radiofrequency Hydro-Injector Device for the Skin around the Eye Area

**DOI:** 10.3390/jcm10122582

**Published:** 2021-06-11

**Authors:** Young-Kyoung Lim, Chang-Jin Jung, Mi-Young Lee, Ik-Jun Moon, Chong-Hyun Won

**Affiliations:** Asan Medical Center, Department of Dermatology, University of Ulsan College of Medicine, Seoul 05505, Korea; ykwh01232@naver.com (Y.-K.L.); juchji630@naver.com (C.-J.J.); myyoung10@naver.com (M.-Y.L.)

**Keywords:** radiofrequency, hyaluronic acid, filler, lateral canthal lines, efficacy, safety

## Abstract

In recent years, variable rejuvenation techniques, such as hyaluronic acid (HA) fillers and radiofrequency (RF) devices, have become popular. We evaluated the RF hydro-injector (RFHI) device that simultaneously delivers both a microneedle intradermal RF treatment and a HA filler injection to overcome the disadvantages of HA filler and RF devices alone. This study aimed to assess the efficacy and safety of the RFHI device for the rejuvenation of the periorbital area, including the lateral canthal lines (LCLs) and the infraorbital area. A total of 24 subjects were enrolled in this study and underwent 2 to 3 treatments using the RFHI device. The investigator’s global assessment of the lateral canthal line (IGA-LCL) and the global esthetic improvement scale (GAIS) were used to evaluate the improvement in the LCL. Cutometer^®^ was used to evaluate the skin’s elasticity, and the Antera 3D image capture system^®^ was used to evaluate the degree of wrinkles, roughness, and pore volume. At the 8 week follow-up after the first treatment session, both the IGA-LCL and GAIS showed significant improvement. The improvement in the wrinkles, roughness, and pore volume, measured by the Antera 3D image capture system^®^, was statistically significant. No serious adverse event was reported. This RFHI device, which delivers both microneedle intradermal RF treatment and HA filler injection, is effective and safe for periorbital rejuvenation.

## 1. Introduction

In recent years, variable rejuvenation techniques are becoming popular among both patients and dermatologists. Aged skin has some distinct features, such as wrinkles, dyschromia, telangiectasia, loss of elasticity, decreased epidermal thickness, and roughness [1]. The growing demand to overcome skin aging has led to the development of a variety of skin rejuvenation technologies, such as radiofrequency (RF) stimulation and hyaluronic acid (HA) filler injection.

RF is a noninvasive rejuvenation technique that is one of the most frequently used treatments in the field of cosmetic dermatology. Non-ablative RF devices generate heat by emitting high-frequency radio waves [2]. The heat induces thermal damage to the collagen, leading to both contraction of the senescent collagen and stimulation of fibroblasts to induce neocollagenesis [3]. Therefore, the RF technique has been used to improve skin laxity and reduce wrinkles on the face, including the periorbital area. Previous research has reported that increased dermal thickness, due to remodeling of the collagen bundles, lasts for months after an RF treatment [4]. Owing to its capability to deliver an exact amount of RF energy at an accurate depth directly into the dermis, fractional RF using a microneedle system is widely used to deliver RF energy to the skin [5].

Soft tissue fillers have been used to fill the wrinkles and folds to achieve facial rejuvenation. Although there are various filler agents available, HA-based fillers are one of the most widely used, owing to their effective and predictable results and relatively favorable safety profile [6]. HA is a natural component of the extracellular matrix of the dermis, with the capacity to hold a large amount of water in the skin. A reduced HA level in aged skin is closely related to volume loss, wrinkle formation, and facial skin laxity [7]. Intradermal HA filler injection has been reported to result in increased hydration of the dermis and the production of collagen [8,9].

Novel combinations of currently available techniques are being actively investigated because skin rejuvenation treatment consisting of multiple modalities generally results in improved efficacy. However, the efficacy and safety of a combination treatment of RF and HA filler injection on skin rejuvenation have not been studied in detail. The RF hydro-injector (RFHI) device is a newly developed investigational device, capable of delivering microneedle RF energy intradermally, while injecting HA filler at the same time. Hence, this study evaluated the efficacy and safety of the RFHI device for the rejuvenation of the periorbital areas, including the lateral canthal lines (LCL) and the infraorbital area.

## 2. Materials and Methods

This study was approved by the institutional review board of the Asan Medical Center (IRB No. 2020-0101) and followed the guidelines of the 1975 Declaration of Helsinki. Signed informed consent was obtained from all patients before their participation in the study.

### 2.1. Study Population

This was a single-center, single-group, open, pilot investigation clinical study designed to evaluate the efficacy and safety of the RFHI device for periorbital areas, including the LCL and infraorbital area. Subjects aged 19–75 years were included in this study, whose LCLs were assessed by the investigator using the investigator’s global assessment of the lateral canthal line (IGA-LCL) [10]. The subjects agreed to use contraception during the study. The exclusion criteria included pregnancy; a history of keloid scars; an infection at the investigational treatment site; hypersensitivity to lidocaine or HA fillers; aesthetic treatments within the past 12 months, including laser skin resurfacing, dermabrasion, filler injections including HA, collagen, and polycaprolactone; botulinum toxin injection, or oral medications including isotretinoin.

### 2.2. Materials

An RFHI device (Agnes Medical Co., Ltd., Gyeonggi-do, Korea), an electric device capable of emitting RF current and injecting hyaluronic filler, was used in this study.

### 2.3. Treatment Procedure

After the screening visit (−2W, −2 weeks), each study subject underwent 2 treatments using the RFHI device at a 2-week interval (W0 and W2). For patients who had not shown improvement on the IGA-LCL after the previous 2 treatments, an additional treatment was performed on W4. The subjects visited the clinic for follow-up assessments at W6 and W8 after the initial treatment.

**Figure 1:**
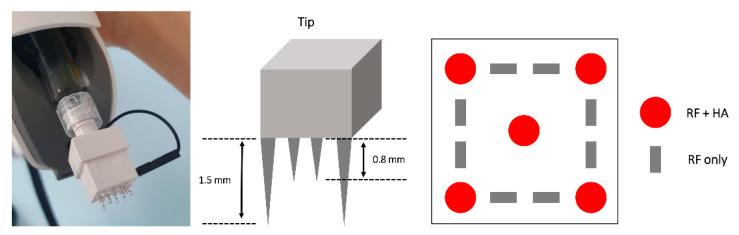
The radiofrequency hydro-injector device consists of five microneedles (34-gauge needle; injection depth, 1.5 mm) delivering both hyaluronic acid filler (HA) and radiofrequency (RF) energy to the deep dermis, and eight microneedles (depth, 0.8mm) delivering RF energy to the superficial dermis.

Before the treatment, topical anesthetic lidocaine cream was applied to the treatment area for 40 minutes. The whole face including the LCL and infraorbital area was treated. After cleansing the skin with chlorhexidine, the RFHI device was applied to the skin. During the RF treatment, the most dominant frequency of the treatment was 1 Mhz, generating a considerable power at 4.0 W and directly delivering energy using 8 microneedles (depth, 0.8 mm). At the same time, each injection contained 0.01 mL of non-cross-linked HA filler (Huons Co., Ltd., Gyeonggi-do, Korea), using an autonomic intradermal injector with 5 microneedles (34-gauge needle; injection depth, 1.5 mm) (Figure 1). At each visit, 3 photographs of the face of each subject were taken.

### 2.4. Efficacy Outcome Measures

Each patient’s face was evaluated before the treatment sessions as the baseline. The LCL improvement was evaluated using IGA-LCL at W0, W2, W4, W6, and W8, as follows: 0, absent; 1, minimal wrinkles within a 1.5 cm radius of the lateral canthus; 2, shallow wrinkles within a 1.5–2.5 cm radius of the lateral canthus; 3, moderately deep wrinkles within a 1.5–2.5 cm radius of the lateral canthus; and 4, long wrinkles exceeding a 2.5 cm radius of the lateral canthus [10]. For subjective evaluation, the global esthetic improvement scale (GAIS) was used at W2, W4, W6, and W8 as follows: −1, worsening; 0, no change; 1, improvement; and 2, much improvement. For objective evaluation, the Cutometer^®^ (Courage and Khazaka, Cologne, Germany) was used to evaluate the improvement in skin elasticity, measured near the LCL on W0, W4, and W8. In addition, the Antera 3D image capture system^®^ (Miravex, Dublin, Ireland) was used to measure the degree of wrinkles, roughness, and pore volume at W0, W2, W4, and W8.

All adverse events (AEs), such as hemorrhage, pain, induration, swelling, redness, and pruritus, in the treated area that occurred after the study were assessed at every visit. The AEs were classified according to their severity using a 3-point grading scale (mild, moderate, and severe).

### 2.5. Statistical Analyses

Data from the IGA-LCL score, GAIS score, skin elasticity values using the Cutometer^®^ (i.e., R2 and R6), and measurements using the Antera 3D image capture system^®^ (i.e., wrinkles, skin roughness, and pore volume) were analyzed for statistical significance through a repeated measures general linear model with numerical predictor variables and a repeated measures ANOVA with categorical predictor variables. (Supplementary Table S1) SPSS version 21.0 (IBM Corp., Armonk, NY, USA) was used for the statistical analyses, considering *p* < 0.05 as statistically significant.

## 3. Results

### 3.1. Subjects and Efficacy Outcomes

**Figure 2:**
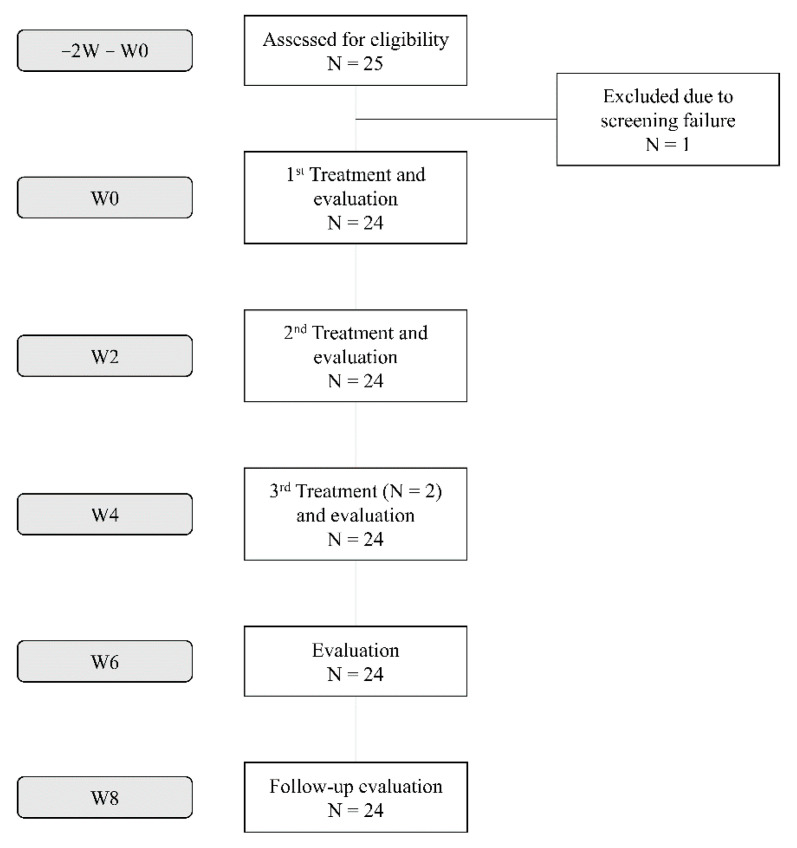
Flowchart of the subjects included in the study.

**Figure 3:**
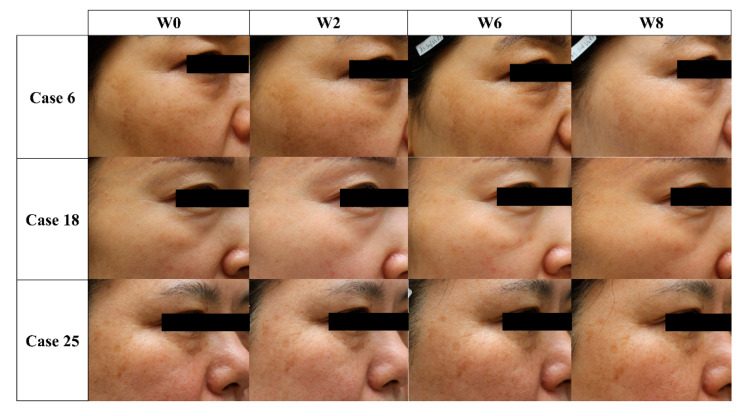
Clinical photographs of three representative cases at week 0, 2, 6, and 8 (W0, W2, W6, and W8). Note the improvements in the wrinkles of the lateral canthal lines and the skin textures after Agnes radiofrequency hydro-injector^®^ (Agnes Medical Co., Ltd., Gyeonggi-do, Korea) treatment.

**Figure 4:**
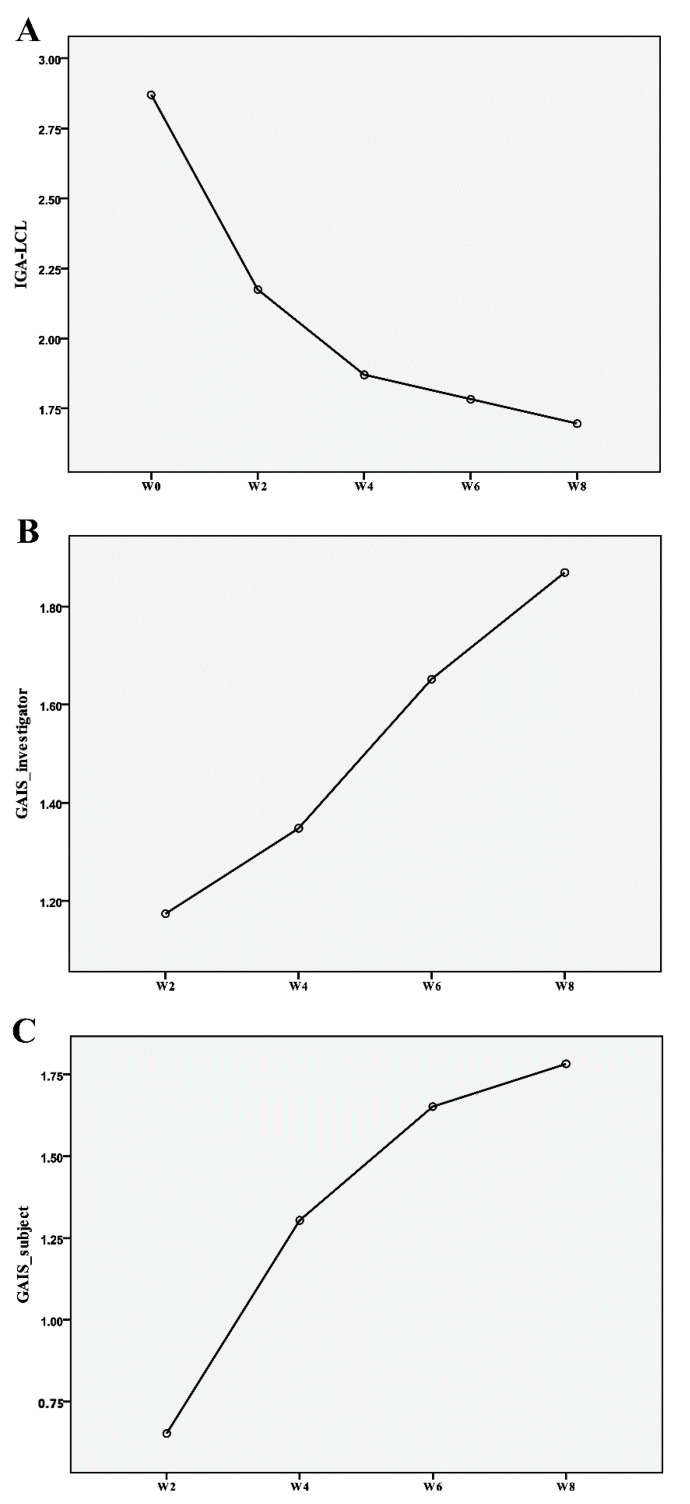
The trends of the (**A**) investigator’s global assessment of the lateral canthal line (IGA-LCL), (**B**) investigator-evaluated, and (**C**) subject-evaluated global esthetic improvement scale (GAIS). The IGA-LCL decreased over time with statistical significance (*p* < 0.001), whereas both investigator-evaluated and subject-evaluated GAIS indicated an increase over time with statistical significance (*p* = 0.001 and *p* < 0.001, respectively).

Of the 25 screened subjects, 24 were included in this study. Although all 24 subjects were treated at W0 and W2, only 2 of the 24 subjects had an additional treatment at W4, owing to their limited improvement on the IGA-LCL after the previous treatments (Figure 2). Moreover, 23 of the 24 subjects were female, and only 1 subject was male. The mean age of the subjects was 50.58 years, ranging from 38 to 63 years. The IGA-LCL decreased over time with statistical significance (*p* < 0.001); the mean ± SD of the IGA-LCL at W0 and W8 were 2.87 ± 0.69 and 1.70 ± 0.70, respectively. The degree of decrease in IGA-LCL was especially noticeable after the first treatment; the additional decreases in the IGA-LCL were also seen at W6 and W8. The investigator-evaluated GAIS indicated an increase over time with statistical significance (*p* = 0.001); the mean ± SD of the investigator-evaluated GAIS at W2 and W8 were 1.17 ± 0.72 and 1.87 ± 0.46, respectively. The subject-evaluated GAIS also indicated a statistically significant increase at each follow-up (*p* < 0.001); the mean ± SD of the subject-evaluated GAIS at W2 and W8 were 0.65 ± 0.65 and 1.78 ± 0.90, respectively. Both the investigator- and subject-evaluated GAIS scores indicated additional increases at W6 and W8, even after the treatment sessions, reporting a value close to score 2 (“much improvement”) (Figure 3 and Figure 4).

### 3.2. Skin Hydration and Elasticity

The Cutometer^®^ was used to evaluate skin hydration and elasticity. R2, referred to as the gross elasticity of the skin including the viscous deformation [11], did not indicate any statistically significant improvement from W0 to W8 (*p* = 0.098). In addition, R6, referred to as the viscoelasticity of the skin indicating the cutaneous water content [12], indicated no statistically significant improvement throughout the study (*p* = 0.205).

### 3.3. Wrinkles, Roughness, and Pore Volume

**Figure 5:**
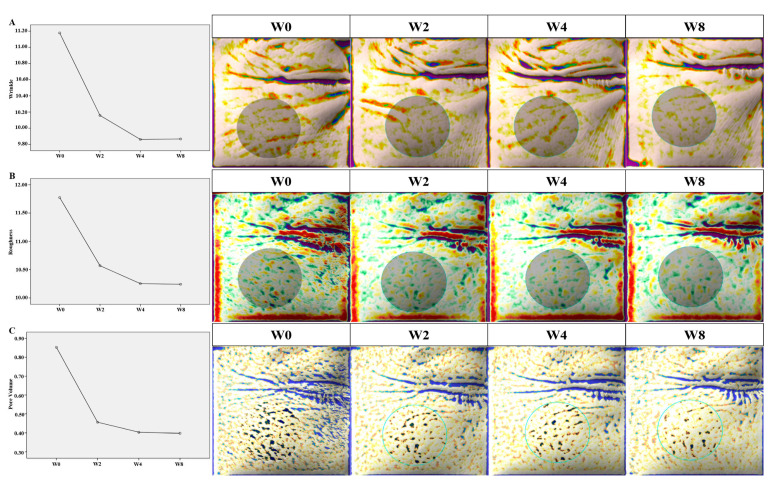
The improvements in wrinkles, roughness, and pore volume measured by the Antera 3D image capture system^®^. (**A**) The wrinkles improved by 11.72% from week 0 (W0) to week 8 (W8) (*p* < 0.001). (**B**) The roughness improved by 13.00% from W0 to W8 (*p* < 0.001). (**C**) The pore volume was reduced by 52.94% from W0 to W8 (*p* < 0.001).

According to the data measured using the Antera 3D image capture system^®^, the wrinkles improved by 9.12% from W0 to W2 after the first treatment session. The degree of improvement in the wrinkles was prominent after the first and second treatments, which was sustained up to W8 without any additional treatment sessions. Overall, the wrinkles improved by 11.72% from W0 to W8. The improvement in the wrinkles was statistically significant throughout the study (*p* < 0.001) (Figure 5A).

The roughness decreased by 10.20% from W0 to W2 after the first treatment session. The degree of decrease in the roughness was noticeable from W0 to W4; the improved texture from W4 to W8 was maintained without any additional treatment sessions. The roughness decreased by 13.00% from W0 to W8 throughout the study, with statistical significance (*p* < 0.001) (Figure 5B).

The pore volume was reduced by 45.88% from W0 to W2 after the first treatment session. After the first treatment session, the degree of reduction in the pore volume was especially striking; the improvement in the pore volume was maintained from W4 to W8, without any additional treatment. In the end, the pore volume was reduced by 52.94% from W0 to W8. The reduction in pore volume was statistically significant throughout the study (*p* < 0.001) (Figure 5C).

### 3.4. Safety Outcomes

Among the 24 patients, treatment-related local AEs occurred in four subjects. After the first treatment session, one patient reported pruritus on the periorbital areas, which resolved spontaneously after 2 days and never relapsed. The other three patients reported localized bruising on the periorbital areas where the treatment was applied, which resolved spontaneously within 2 weeks. No serious AEs were reported. There was no dropout or withdrawal because of AEs in this study.

## 4. Discussion

Soft tissue fillers and RF devices are widely used modalities for skin rejuvenation. Owing to their favorable outcomes and relatively few AEs, HA fillers are the most widely used among the available filler materials. HA is a component of the extracellular matrix of the skin, with a strong water-binding capacity, which facilitates skin hydration to improve the viscoelasticity and firmness of the skin [13]. Owing to its minimal immunogenicity and degradability by hyaluronidase, HA fillers have a relatively low rate of serious AEs [14]. Even though HA fillers have an immediate effect of volume restoration, they have a relatively short duration of action owing to their biodegradability, which requires frequent injections for maintenance [15].

Using nonionized electromagnetic waves at a frequency between 3 KHz and 300 GHz, RF is an effective treatment for skin tightening [16]. RF treatment generates high thermal energy through electrical resistance of the skin, inducing immediate tissue contraction with subsequent neocollagenesis and dermal remodeling for skin tightening [3,4]. Because a particular chromophore is not the target of RF treatment, RF treatment can be applied safely for all skin types [17]. However, RF treatment has a limited capacity of immediate volume restoration, requiring repetitive treatments for skin tightening and volume restoration [18].

Several studies have evaluated the effect of a combination treatment of both modalities to overcome the individual limitations of HA filler injections and RF treatment [19,20,21]. England et al. [19] reported no adverse reactions or impact on the residence time of various fillers after multiple passes of RF treatment directly over filler-injected skin in an animal model. Goldman et al. [20] suggested the safety profile of RF treatment applied immediately after HA gel implantation in a human model. Choi et al. [21] reported synergistic effects of a combination therapy of intradermal RF and HA filler to reduce nasolabial fold wrinkles.

This study demonstrated an improvement in the wrinkles of the lateral canthus, roughness, and pore volume of the skin after two or three sessions of RFHI treatment. The improvement in the wrinkles of the lateral canthus was shown through both subjective and objective measurements—at least one stage decrease in the mean IGA-LCL, and an 11.72% improvement in wrinkles as evaluated by the Antera 3D image capture system^®^. With regard to wrinkles of the lateral canthus, both the investigator- and subject-evaluated GAIS indicated statistically significant improvements throughout the study. In addition, the improvement in roughness and pore volume of the skin was proven to be 13.00% and 52.94%, respectively. The improvement after two or three sessions of treatment using the RFHI device was maintained for at least 4 to 6 weeks.

The RFHI device delivers RF energy directly to the dermis, before injecting HA filler with the same needle electrodes. Choi et al. [21] proposed that the formation of “autologous containment collagen canals”, induced by microneedle RF treatment, could provide protection from external oxygen radicals and hold the injected HA filler so it does not spread away from the targeted area. Thus, a combination treatment of microneedle intradermal RF and HA filler injection could have several advantages. First, RF treatment could stimulate the production of glycosaminoglycans, which are hydrophilic molecules with high water-binding capacity [22]. Therefore, applying intradermal RF before HA filler injection could have synergistic effects to improve skin hydration. Second, RF treatment reduces elastotic materials by reorienting elastic fibers and stimulating new collagen formation, which can be helpful to extend the duration of the effects of HA filler [23]. Third, although injecting HA filler with microneedles allows for an even mid-dermal distribution of HA filler to achieve a safe and effective outcome, microneedling itself can stimulate epidermal HA synthesis through upregulated hyaluronan synthase expression, and increased collagen and elastic fiber synthesis, improving wrinkles and scars [24,25]. Fourth, because injected filler can break down and spread away faster in mobile areas, including the periorbital areas, RF treatment before HA filler injection may provide long-lasting effects to improve wrinkles through RF-induced neocollagenesis, even after the degradation of the injected filler [18]. Finally, RF treatment before HA filler injection can lower the possibility of adverse effects, in that not only does HA filler have minimal immunogenicity, but also RF energy does not affect the HA filler [18]. In addition, previous treatment with microneedle intradermal RF may induce the coagulation of vessels in the area where RF is applied, which can reduce bruising and the risks of vascular events after intradermal HA filler injection [21].

With regard to hydration and elasticity of the skin, the values of R2 and R6 measured by the Cutometer^®^ indicated no significant differences from W0 to W8. R2 is referred to as the gross elasticity of the skin in the range of 0 to 1, and is the main parameter to evaluate skin elasticity and aging [11]. R6 is referred to as the viscoelasticity of the skin, indicative of skin hydration [12]. Because the Cutometer^®^ measures superficial cutaneous changes to assess elasticity and hydration of the skin, it would be hard to evaluate the dermal changes in this study [26]. In addition, the measurements of skin elasticity and hydration might be affected by seasonal variations, because this study spanned from summer to winter [27]. Therefore, further studies designed to exclude the effect of seasonal variations using objective assessment modalities, including skin biopsy, to analyze collagen and elastic fibers would be beneficial to accurately evaluate the improvement in skin elasticity after RFHI treatment.

This study has some limitations. Because this study was not designed as a split-face study, due to ethical issues, we could not assess the objective synergistic effect of combination treatment of intradermal RF and HA filler injection compared with solitary RF treatment or HA filler injection. Another limitation was the relatively small sample size. In addition, the duration of follow-up was not long enough to evaluate the long-term effect of the combination treatment. Further studies designed as split-face studies, with a large sample size and a long duration of follow-up, are needed to solidify the synergistic effect and safety of combination treatment of intradermal RF and HA filler injection.

## 5. Conclusions

The RFHI device, a newly developed investigative device to deliver a combination treatment of microneedle intradermal RF and HA filler injection, was proven to be an effective and safe modality for skin rejuvenation, especially in periorbital areas, including the LCL and infraorbital area. Also, the results of this study indicate a possible use of RF in combination with intradermal injection of several other agents, for either medical or cosmetic purposes, in order to benefit from their synergistic effects.

## Data Availability

The data that support the findings of this study are available from the corresponding author upon reasonable request.

## Supplementary Materials

The following are available online at https://www.mdpi.com/article/10.3390/jcm10122582/s1, Table S1: The analysis of variance table.

## References

[1] Sumino H., Ichikawa S., Abe M., Endo Y., Ishikawa O., Kurabayashi M. (2004). Effects of aging, menopause, and hormone replacement therapy on forearm skin elasticity in women. J. Am. Geriatr. Soc..

[2] Carruthers J., Carruthers A. (2007). Shrinking upper and lower eyelid skin with a novel radiofrequency tip. Dermatol. Surg..

[3] Elsaie M.L., Choudhary S., Leiva A., Nouri K. (2010). Nonablative radiofrequency for skin rejuvenation. Dermatol. Surg..

[4] Zelickson B.D., Kist D., Bernstein E., Brown D.B., Ksenzenko S., Burns J., Kilmer S., Mehregan D., Pope K. (2004). Histological and ultrastructural evaluation of the effects of a radiofrequency-based nonablative dermal remodeling device: A pilot study. Arch. Dermatol..

[5] Hantash B.M., Renton B., Berkowitz R.L., Stridde B.C., Newman J. (2009). Pilot clinical study of a novel minimally invasive bipolar microneedle radiofrequency device. Lasers Surg. Med..

[6] Philipp-Dormston W.G., Bergfeld D., Sommer B.M., Sattler G., Cotofana S., Snozzi P., Wollina U., Hoffmann K.P.J., Salavastru C., Fritz K. (2017). Consensus statement on prevention and management of adverse effects following rejuvenation procedures with hyaluronic acid-based fillers. J. Eur. Acad. Dermatol. Venereol..

[7] Gerth D.J. (2015). Structural and volumetric changes in the aging face. Facial Plast. Surg..

[8] Wang F., Garza L.A., Kang S., Varani J., Orringer J.S., Fisher G.J., Voorhees J.J. (2007). In vivo stimulation of de novo collagen production caused by cross-linked hyaluronic acid dermal filler injections in photodamaged human skin. Arch. Dermatol..

[9] Seok J., Hong J.Y., Choi S.Y., Park K.Y., Kim B.J. (2016). A potential relationship between skin hydration and stamp-type microneedle intradermal hyaluronic acid injection in middle-aged male face. J. Cosmet. Dermatol..

[10] Kane M.A., Blitzer A., Brandt F.S., Glogau R.G., Monheit G.D., Narins R.S., Paty J.A., Waugh J.M. (2012). Development and validation of a new clinically-meaningful rating scale for measuring lateral canthal line severity. Aesthet Surg. J..

[11] Ahn S., Kim S., Lee H., Moon S., Chang I. (2007). Correlation between a Cutometer and quantitative evaluation using Moire topography in age-related skin elasticity. Ski. Res. Technol..

[12] Akhtar N., Zaman S.U., Khan B.A., Amir M.N., Ebrahimzadeh M.A. (2011). Calendula extract: Effects on mechanical parameters of human skin. Acta Pol. Pharm..

[13] Kerscher M., Bayrhammer J., Reuther T. (2008). Rejuvenating influence of a stabilized hyaluronic acid-based gel of nonanimal origin on facial skin aging. Dermatol. Surg..

[14] Kim J.E., Sykes J.M. (2011). Hyaluronic acid fillers: History and overview. Facial Plast. Surg..

[15] el-Domyati M., el-Ammawi T.S., Medhat W., Moawad O., Brennan D., Mahoney M.G., Uitto J. (2011). Radiofrequency facial rejuvenation: Evidence-based effect. J. Am. Acad. Dermatol..

[16] Kim J.E., Chang S., Won C.H., Kim C.H., Park K.H., Choi J.H., Lee M.W. (2012). Combination treatment using bipolar radiofrequency-based intense pulsed light, infrared light and diode laser enhanced clinical effectiveness and histological dermal remodeling in Asian photoaged skin. Dermatol. Surg..

[17] Akita H., Sasaki R., Yokoyama Y., Negishi K., Matsunaga K. (2014). The clinical experience and efficacy of bipolar radiofrequency with fractional photothermolysis for aged Asian skin. Exp. Dermatol..

[18] Kim H., Park K.Y., Choi S.Y., Koh H.J., Park S.Y., Park W.S., Bae I.H., Kim B.J. (2014). The efficacy, longevity, and safety of combined radiofrequency treatment and hyaluronic Acid filler for skin rejuvenation. Ann. Dermatol..

[19] England L.J., Tan M.H., Shumaker P.R., Egbert B.M., Pittelko K., Orentreich D., Pope K. (2005). Effects of monopolar radiofrequency treatment over soft-tissue fillers in an animal model. Lasers Surg. Med..

[20] Goldman M.P., Alster T.S., Weiss R. (2007). A randomized trial to determine the influence of laser therapy, monopolar radiofrequency treatment, and intense pulsed light therapy administered immediately after hyaluronic acid gel implantation. Dermatol. Surg..

[21] Choi S.Y., Lee Y.H., Kim H., Koh H.J., Park S.Y., Park W.S., Bae I.H., Park K.Y., Kim B.J. (2014). A combination trial of intradermal radiofrequency and hyaluronic acid filler for the treatment of nasolabial fold wrinkles: A pilot study. J. Cosmet. Laser Ther..

[22] Augustyniak A., Rotsztejn H. (2016). Nonablative radiofrequency treatment for the skin in the eye area: Clinical and cutometrical analysis. J. Cosmet. Dermatol..

[23] Hantash B.M., Ubeid A.A., Chang H., Kafi R., Renton B. (2009). Bipolar fractional radiofrequency treatment induces neoelastogenesis and neocollagenesis. Lasers Surg. Med..

[24] Tammi R., Pasonen-Seppanen S., Kolehmainen E., Tammi M. (2005). Hyaluronan synthase induction and hyaluronan accumulation in mouse epidermis following skin injury. J. Investig. Dermatol..

[25] Aust M.C., Fernandes D., Kolokythas P., Kaplan H.M., Vogt P.M. (2008). Percutaneous collagen induction therapy: An alternative treatment for scars, wrinkles, and skin laxity. Plast Reconstr. Surg..

[26] Jeon I.K., Chang S.E., Park G.H., Roh M.R. (2013). Comparison of microneedle fractional radiofrequency therapy with intradermal botulinum toxin a injection for periorbital rejuvenation. Dermatology.

[27] Lee Y.J., Kim H.T., Lee Y.J., Paik S.H., Moon Y.S., Lee W.J., Chang S.E., Lee M.W., Choi J.H., Jung J.M. (2020). Comparison of the effects of polynucleotide and hyaluronic acid fillers on periocular rejuvenation: A randomized, double-blind, split-face trial. J. Dermatolog. Treat..

