# Supplementary material for: The Evaluation of Efficacy and Safety of A Radiofrequency Hydro-Injector Device for the Skin around the Eye Area
Source: J Clin Med. 2021 Jun 11;10(12):2582. doi: 10.3390/jcm10122582 (PMC8230740; doi:10.3390/jcm10122582)
Supplement: Supplementary file 1 [file jcm-10-02582-s001.zip › jcm-1199526-supplementary.pdf]

Supplementary table SI.

	df	Mean square	F	p-value
**IGA-LCL**				
Within-subject effect				
Greenhouse-Geisser	2.917	7.197	36.626	0.000
Huynh-Feldt	3.410	6.155	36.626	0.000
Within-subject contrast				
Linear	1	17.257	78.382	0.000
**GAIS-Investigator**				
Within-subject effect				
Greenhouse-Geisser	2.134	3.112	8.298	0.001
Huynh-Feldt	2.370	2.802	8.298	0.000
Within-subject contrast				
Linear	1	6.576	27.962	0.000
**GAIS-Subject**				
Within-subject effect				
Greenhouse-Geisser	2.275	7.759	16.997	0.000
Huynh-Feldt	2.551	6.919	16.997	0.000
Within-subject contrast				
Linear	1	16.078	32.686	0.000
**Wrinkle**				
Within-subject effect				
Greenhouse-Geisser	2.357	11.856	8.278	0.000
Huynh-Feldt	2.643	10.572	8.278	0.000
Within-subject contrast				
Linear	1	21.436	12.197	0.002
**Roughness**				
Within-subject effect				
Greenhouse-Geisser	2.214	17.114	11.025	0.000
Huynh-Feldt	2.460	15.402	11.025	0.000
Within-subject contrast				
Linear	1	28.949	15.483	0.001
**Pore volume**				
Within-subject effect				
Greenhouse-Geisser	1.367	2.514	13.957	0.000
Huynh-Feldt	1.424	2.413	13.957	0.000
Within-subject contrast				
Linear	1	2.409	16.852	0.000
